# Improved weighting in particle filters applied to precise state estimation in GNSS

**DOI:** 10.3389/frobt.2022.950427

**Published:** 2022-08-11

**Authors:** Simone Zocca, Yihan Guo, Alex Minetto, Fabio Dovis

**Affiliations:** Department of Electronics and Telecommunications (DET), Politecnico di Torino, Turin, Italy

**Keywords:** bayesian estimation, global navigation satellite system, particle filter, positioning and navigation, sequential Monte Carlo (SMC)

## Abstract

In the last decades, the increasing complexity of the fusion of proprioceptive and exteroceptive sensors with Global Navigation Satellite System (GNSS) has motivated the exploration of Artificial Intelligence related strategies for the implementation of the navigation filters. In order to meet the strict requirements of accuracy and precision for Intelligent Transportation Systems (ITS) and Robotics, Bayesian inference algorithms are at the basis of current Positioning, Navigation, and Timing (PNT). Some scientific and technical contributions resort to Sequential Importance Resampling (SIR) Particle Filters (PF) to overcome the theoretical weaknesses of the more popular and efficient Kalman Filters (KFs) when the application relies on non-linear measurements models and non-Gaussian measurements errors. However, due to its higher computational burden, SIR PF is generally discarded. This paper presents a methodology named Multiple Weighting (MW) that reduces the computational burden of PF by considering the mutual information provided by the input measurements about the unknown state. An assessment of the proposed scheme is shown through an application to standalone GNSS estimation as a baseline of more complex multi-sensors, integrated solutions. By relying on the *a-priori* knowledge of the relationship between states and measurements, a change in the conventional PF routine allows performing a more efficient sampling of the posterior distribution. Results show that the proposed strategy can achieve any desired accuracy with a considerable reduction in the number of particles. Given a fixed and reasonable available computational effort, the proposed scheme allows for an accuracy improvement of the state estimate in the range of 20–40%.

## 1 Introduction

From a general perspective, many problems in Artificial Intelligence (AI) and robotics applications may depend on positioning and navigation data (e.g., path planning, autonomous obstacle perception, collision avoidance). A growing number of AI services indeed infer traffic conditions and support situational awareness and decision making by leveraging such information. In these context, multiple agents aim at solving state estimation problems by processing proprioceptive and exteroceptive input measurements affected by noise. Nowadays, AI gathers a number of methods to deal with similar applications. Such methods were historically part of different disciplines like information, estimation and control theory. Besides, many tools developed to solve for localization and tracking belong to probability theory such as Bayesian inference and associated algorithms (Luger, 2005; Russell and Norvig, 2010). Bayesian algorithms relying on Hidden Markov Models (HMM) are extensively exploited in predictive filtering problems applied to state estimation (Poole and Mackworth, 2017). By supporting service robotics, autonomous vehicles and Intelligent Transportation Systems (ITS) at a larger extent, navigation systems handle discrete-time HMM for object tracking and state estimation that rely on a set of observable measurements, e.g., range, bearing or heading (Ristic et al., 2004; Gustafsson et al., 2002).

To this aim, a variety of algorithms belonging to the classes of Kalman Filter (KF) and Sequential Monte Carlo (SMC) like the Particle Filter (PF) are exploited in modern Global Navigation Satellite System (GNSS) receivers to infer its Position Time Velocity (PVT). KF estimation is widely used due to its lower computational load w.r.t. the other approaches. However, in many complex scenarios, KF solutions are sub-optimal when the errors on the measurements cannot be modeled through normal distributions. Furthermore, when dealing with highly non-linear system models, the performance of Extended Kalman Filter (EKF) is limited by the approximations caused by the linearization of the problem. Unlike KF-based solutions, PF can deal with any given error density as well as with non-linear system models. This feature eases the implementation of a variety of hybridization schemes that combine heterogeneous measurements for enhancing GNSS such as sensor fusion and collaborative localization (Georgy et al., 2010; Minetto et al., 2020; Shen et al., 2019; Xia et al., 2020). In these applications, it has been shown that PF is able to provide improved performance, but at the cost of a non-negligible computational complexity, especially for high cardinality of the state space (Minetto et al., 2019). Indeed, the number of particles needed to accurately represent the a-posteriori densities exponentially increases with the cardinality of the state space (Poterjoy, 2016). In applications with loose constraints on computational or power-consumption, PFs may mitigate sub-optimalities of other approaches, as in (Zocca et al., 2021) where the filter is adapted to a non stationary statistics of the input measurements.

Furthermore, SMC methods and in particular PF has been historically recognized as a tool for AI (Thrun, 2002; Russell and Norvig, 2010; Poole and Mackworth, 2017; Sutton and Barto, 2018), as also shown in (Carrera Villacrés et al., 2019), where a PF is used as a global search method in reinforcement learning; while (Ma et al., 2020) uses instead a set of particles to maintain an approximate latent state distribution in recurrent neural networks. In fact, among its many applications, a possible view of PF is that of a search mechanism that machine learning algorithms can leverage, similarly to a gradient descend algorithm.

Many variants of the PF have been proposed in the literature to deal with its computational burden and main theoretical limitations (Ristic et al., 2004). For instance, if the state model contains a linear sub-structure subject to Gaussian noise, Rao-Blackwellized PF (also denoted in the literature as Marginalized PF) allows to solve any linear sub-structure through KF, thus reducing the number of dimensions sampled by the PF (Schon et al., 2005; Zhou et al., 2019). PF exploiting an adaptive number of particles was also proposed to overcome the complexity issue (Closas and Fernández-Prades, 2011). Several works aimed at mitigating particles degeneracy using hybrid filtering schemes such as Unscented Particle Filter (UPF) and Auxiliary Particle Filter (APF) (Yu et al., 2020; Song et al., 2021).

Contrary to the remarkable availability of PF algorithms and variants, application-specific optimizations appear lacking in the literature and few works address optimizations in the weighting of the particles. Despite the advantage of the PF lies in its ability to deal with heterogeneous statistics of measurements and non-linear systems, the presented optimization strategy can be employed regardless of the scenario and is derived from the mathematical relationship between the quantities involved in the problem. For this reason, it is important to stress that the technique presented here is valid not only for any kind of hybrid scheme integrating additional measurements to GNSS, but to other estimation problems as well. In order to simplify the discussion and focus solely on the proposed technique, we address a baseline scenario, where the PF is dealing with GNSS only. In any case, the weighting strategy we propose can be extended to more complex receiver architecture, where the PF would be more suited and advantageous, without any loss of validity. In light of this, this paper presents a solution based on Sequential Importance Resampling (SIR) PF, through the following contributions:

• A strategy to optimize the use of particles which leverages the information carried by different subsets of input measurements;
• A statistical derivation of the advantage brought by the proposed technique, along with a numerical example for a more direct and visual understanding of the proposed approach;
• An experimental assessment using real GNSS measurements to demonstrate the accuracy improvement provided by the proposed approach in real applications.

### 1.1 Theoretical background

This section recalls some theoretical background on state estimation via SIR PF that is needed before introducing the main contributions of our work. A more in-depth discussion of SMC methods can be found in (Cappé et al., 2007; Elfring et al., 2021) Despite our work focusing on the implementation of PF for GNSS positioning, we present in this section a general terminology and notation that is valid for state estimation problems to a larger extent.

#### 1.1.1 Recursive bayesian state estimation

In general, the problem of estimating hidden states can be modeled using a discrete-time HMM. Following the Markov assumption, the sequence of states is modeled by a Markov chain. Therefore, the probability of the current state ***θ***_*k*_ is only based on the previous one ***θ***_*k*−1_ and conditionally independent of all other earlier states. In other words, the current state only depends on the previous one, because the latter summarizes the entire history. Likewise, the current measurements **z**_*k*_ only depends on the current state ***θ***_*k*_. Thanks to these properties, the system can be described simply with the following items:

• ***θ***_*k*_, is a stochastic *state space vector* of the hidden states (those to be estimated);
• ***θ***_*k*_ = **f**(***θ***_*k*−1_, **w**_*k*−1_), is a function describing the discrete-time *state space transition*, which also accounts for process noise **w**_*k*−1_;
• **z**_*k*_ = [ z_1,*k*_ … z_*M*,*k*_ ], is a vector of *M* synchronous and independent *input measurements*, also referred to as *observables*;
• **z**_*k*_ = **h**(***θ***_*k*_, **v**_*k*_), which is called the *observables-states function* and models the relationship between observations and hidden state space, also accounting for observation noise **v**_*k*_.

Bayesian filters are able to exploit the *a-priori* knowledge of the state space transition function **f**(***θ***_*k*−1_, **w**_*k*−1_) to estimate the state space vector $\hat{\theta }$ which maximizes the a-posteriori probability of the observations **z**_*k*_ (Brown and Hwang, 2012). The inference of state variables is iteratively performed through a cyclic prediction-update approach, which allows to successfully mitigate the effect of noisy measurements. A fundamental characteristic of the PF is that, differently from other classes of Bayesian estimators, it does not impose strict constraints on the items of the Bayesian estimation problem (Arulampalam et al., 2002).

#### 1.1.2 State estimation using SIR PF

**FIGURE 1:**
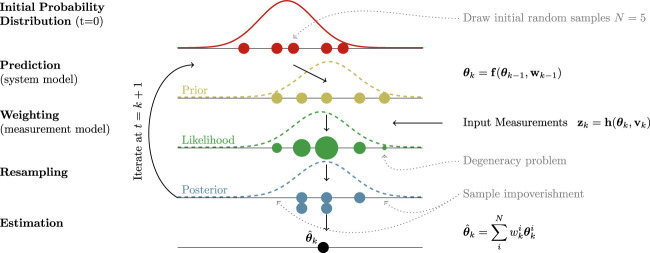
Simple scheme of the routine of a SIR PF architecture implementing Bayesian state space estimation. In each stage, particles (depicted with circles) are represented with a different color. The corresponding name of the stage is on the left side, while the right side is devoted to present some important equations governing the system and highlighting the main issues that PF face. The radius of particles is proportional to their weight.

The main idea of PF is to use sets of random samples (called particles) to represent a Probability Density Function (PDF). In short, PF uses Bayes’ theorem to obtain a discrete approximation of the probability density function of the state space (Posterior) by combining statistical knowledge of earlier states (Prior) and current measurements (Likelihood). Following the state space transition model, the posterior becomes the prior at the new iteration, and so on.The main stages of a SIR PF routine are shown in the scheme of Figure 1. On the first epoch, the filter is initialized by sampling a set of *N* particles from an initial distribution and assigning initial importance weights (Cappé et al., 2007). Each *i*th particle $\theta_{k}^{i}$ is a possible realization of the state space vector ***θ***_*k*_. Subsequently in the prediction stage, each of the *N* particles is propagated forward according to the state space transition model $\theta_{k}^{i}=f\left(\theta_{k-1}^{i},w_{k-1}\right)$ (Arulampalam et al., 2002). This step is closely related to the prediction of non-SMC estimators, i.e. KFs, which instead operate on a single prediction of the state space.Afterwards, the vector of nominal measurements **z** ^*i*^ is computed for each particle. This steps consists in computing, using the state-observables function **h**, what would be the nominal measurements obtained by each particle, given their states. Then, weights are computed by relying on probability density models, $p\left(z_{m,k}|\theta_{k}^{i}\right)$ , w.r.t. the input measurements. We first define

$${\bar{z}}_{m,k}^{i}=z_{m,k}-z_{m,k}^{i}$$ (1)

which accounts for the misclosure between the observables z_*m*,*k*_ and the corresponding nominal quantity for the *i*th particle, $z_{m,k}^{i}$ . In words, ${\bar{z}}_{m,k}^{i}$ represents the difference between what one of the current input measurements and what each particles would measure without accounting for observation noise. Since the state space transition function is used as proposal density, the unnormalized weights for statistically independent measurements can be computed as

$${\tilde{w}}_{k}^{i}=w_{k-1}^{i}L\left(z_{k}|\theta_{k}^{i}\right)=w_{k-1}^{i}\overset{M}{\underset{m=1}{\prod }}p\left({\bar{z}}_{m,k}^{i}\right)$$ (2)

This means that $p\left({\bar{z}}_{m,k}^{i}\right)$ represents the probability that the *i*th particle would have measured the corresponding input observable. Therefore, the likelihood for each particle L represents the probability that it has observed the entire set of input measurements.Since the aim of PF is obtain a discrete representation of a continuous PDF, weights are then normalized according to

$$w_{k}^{i}=\frac{{\tilde{w}}_{k}^{i}}{\sum_{i=1}^{N}{\tilde{w}}_{k}^{i}}$$ (3)

so that they sum up to one.

A common problem is that, after a few iterations, there is an increase in variance of particles due to the presence of process noise (as time is propagated forward in the prediction step, the uncertainty on the system increases). From a practical perspective, this means that many particles have weights close to zero, and therefore they do not contribute in representing the posterior. This phenomenon is known as the *degeneracy problem* (Arulampalam et al., 2002).

To solve this problem, the concept of resampling has been introduced (Rubin, 1988). The purpose of this step is to draw a new set of *N* particles based on the starting set. In particular, each particles has a probability of being chosen that is proportional to its weight. As a consequence, the particles with small weight are very likely to not be chosen and not appear in the new set, while particles with large weight are very likely to be chosen and can appear multiple times. While resampling effectively solves the problem of degeneracy by getting rid of many particles with low weights, it introduces a new problem known as *sampling impoverishment*. Since some particles disappear from the resampled set, the target PDF is sampled in fewer points, meaning the knowledge of the value of the PDF in such points is lost. However, this step is crucial as it essentially balances the growth of variance in particles.

The most basic resampling strategy, which we will consider for the remainder of this discussion for the sake of simplicity, is to perform resampling at each iteration. A more efficient alternative strategy to limit the computational load of this stage would be to first compute the effective number of particles as

$$N_{e}=\frac{1}{\sum_{i=1}^{N}{\left(w^{i}\right)}^{2}}$$ (4)

and choose to perform resampling when this value drops below a certain threshold. Since the probability of resampling is proportional to the weight of each particle, the weights of the newly drawn particles are all set to $w^{i}=\frac{1}{N}$ . Therefore, given our strategy to resample at every iteration, (2) can be simplified by neglecting the weight $w_{k-1}^{i}$ from the previous iteration. Because of its effect on the computational load, many efficient resampling strategies have been proposed and analysed in the literature (Bolić et al., 2004; Li et al., 2015), but a more detailed discussion of the resampling stage is out of the scope of this work. After the prediction and correction steps have been performed, the cloud of particles now represents a discrete estimate of the posterior distribution which we are interested in. The output estimate can be obtained as a weighted sum of the particles

$${\hat{\theta }}_{k}=\overset{N}{\underset{i=1}{\sum }}w_{k}^{i}\theta_{k}^{i}$$ (5)

which replaces the integral operation on continuous probability functions.

## 2 Materials and methods

### 2.1 Multiple weighting (MW) approach

When applying legacy PF to estimation problem, the entire set of input measurements to compute a single weight for each particle as in (2). However, in some cases, not all input measurements are related to all observables through the measurement model. In particular, it can be that different classes of measurements are related to only non-overlapping subsets of the state space. Standard PFs mix all the available information into a single weight, which gives an overall likelihood across the whole state space, but a more intelligent use of resources is possible by leveraging the knowledge of the state-observables relationship.

In such cases, similar measurements can be grouped into *J* subsets of the observables **z**_(*j*)_, from which multiple weights *w*_(*j*)_ are derived to estimate the corresponding sub-spaces of the state vector ***θ***_(*j*)_. Subset indexes are noted using round brackets, while time indexes have been dropped from the remainder of the discussion for the sake of readability. In order to characterize the *information diversity* from dissimilar measurements, multiple observables-state functions can be defined

$$z_{\left(j\right)}=h_{\left(j\right)}\left(\theta_{\left(j\right)},v_{\left(j\right)}\right)\text{ where }z_{\left(j\right)}\subset z.$$ (6)

By leveraging the aforementioned simplification thanks to the resampling strategy considered, (2) can be rewritten for the *j*th independent weight is computed as

$${\tilde{w}}_{\left(j\right)}^{i}=L\left(z_{\left(j\right)}|\theta^{i}\right)=\overset{M_{\left(j\right)}}{\underset{m=1}{\prod }}p\left({\bar{z}}_{m}^{i}\right)$$ (7)

where *M*_(*j*)_ is the number of measurements in vector **z**_(*j*)_. A different resampling strategy would not allow for the simplification shown in (7), as the weight from the previous epoch would still appear in (7), but the proposed strategy would still be valid, as this is simply introduced to simplify notation in our discussion. Equivalently, the state estimate from (5) is also modified as

$${\hat{\theta }}_{\left(j\right)}=\overset{N}{\underset{i=1}{\sum }}w_{\left(j\right)}^{i}\theta_{\left(j\right)}^{i}$$ (8)

so that the different subsets are estimated independently using their corresponding weights. In this architecture, the resampling stage can be performed fully independently on the different subsets using the corresponding weights to draw the resampled subsets. Eventually, the estimated subsets are obtained according to (8) and then merged together, as well as the subsets of each particle with the same index *i*. Since we are interested in approximating a discrete probability density, the indexing of the particles does not influence the output estimate. The outcome of (8) only depends on values and weights of particles. It is important to highlight that while the sampling of the two subsets is performed independently, position and velocity are still tied in the dynamic model and used jointly in the prediction step, as one is the derivative of the other, and hence the two quantities cannot be fully decoupled. The proposed technique performs the split only during the sampling and resampling stages to leverage the information diversity to reduce the dimensionality of the problem.

An alternative solution could be to distribute the estimation over multiple filters instead, with each one devoted to the estimation of a subset of states, as developed in (Djuric et al., 2007) and (Djurić and Bugallo, 2013). This solution would still require the filters to share information as different subsets of states can still be related in the system model (e.g. prediction of position at next epoch depends on the velocity), and hence comes at the cost of a more complex architecture w.r.t. the solution presented here. The state propagation cannot be performed independently for the defined subsets, differently the advantage of model-based Maximum-A-Posteriori (MAP) estimation is unexploited, turning into a different estimation paradigm, i.e., Maximum Likelihood (ML).

A related problem was discussed in (Davey et al., 2011), when integrating asynchronous measurements from dissimilar sensors, and was solved by proper modifications of the resampling stage. In our case, measurements are dissimilar but collected synchronously. When measurement information is merged into a single weight, the likelihood of each subset of states is lost and only an overall likelihood of each particle is retained in the standard PF approach. For this reason, the problem has to be addressed before the resampling stage and the mentioned approach cannot be applied to this scenario.

Local PFs (Rebeschini and Van Handel, 2015) have also been addressed in distributed, multi-sensor tracking techniques (Closas and Bugallo, 2012; Maskell et al., 2006) in order to maintain a dimension-free approximation error. This approach is possible in state space models where block of observations are conditionally independent given the hidden state and only depend on separate components of the hidden state.

Furthermore, the proposed solution differs from classic Rao-Blackwellized PF (Schon et al., 2005), as all subsets of the state space are estimated through PF, thus preserving the fundamental properties of SMC methods.

### 2.2 Theoretical proof

**FIGURE 2:**
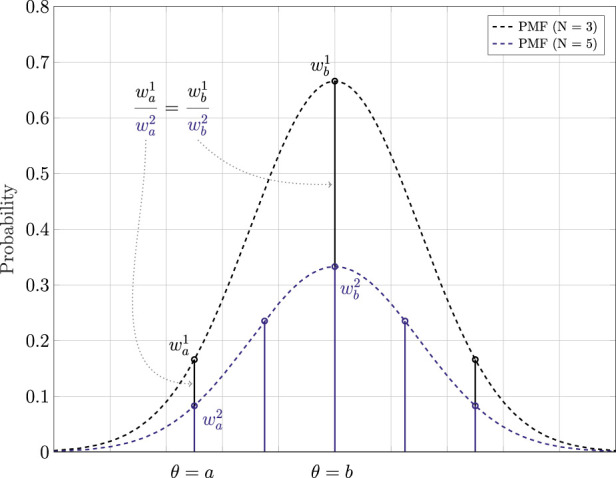
One dimensional example of using a sampling strategy to generate PMFs to approximately represent a PDF. Each PMF with a different number of particles *N* is shown with a different color. For the example, subscripts refer to the value at which sampling is performed.

In the state estimation problem, the original posterior distribution of the states is a continuous form PDF *f*_***θ***_. SMC methods leverages a large number of particles to form a discrete distribution which approximates the original continuous distribution. The normalization step is then taken to ensure the summation of the Probability Mass Function (PMF) is always equal to 1 as from (3). Therefore, if we use different numbers of particles to represent the same continuous distribution, the PMFs will be different, as can be seen in Figure 2.Let’s assume that we have a state space vector containing two independent states ***θ*** = [ *θ*_1_ *θ*_2_ ]; we want to estimate them using both PF and Multi Weight Particle Filter (MW-PF) and compare the solutions. Let’s consider as an example the estimation of the first state *θ*_1_. Given a fixed number of particles, we want to determine which discrete marginal distribution of *θ*_1_ is more accurate between those obtained with PF or MW-PF. For this discussion, we will consider a set of *N* particles (indexed using superscript) and focus more specifically on two particles with given values ***θ*** ^***1***^ = [ *a c* ] and ***θ*** ^***2***^ = [ *b* *d* ].First, we consider what happens when employing MW-PF. We define the subsets as *θ*_(1)_ = [ *θ*_1_ ] and *θ*_(2)_ = [ *θ*_2_ ]. Since in this case the subsets only contain one state, vector notation is not used. The corresponding independent weights are computed using (7). For the two particles with values $\theta_{\left(1\right)}^{1}=a$ and $\theta_{\left(1\right)}^{2}=b$ , we want

$$\frac{w_{\left(1\right)}^{1}}{w_{\left(1\right)}^{2}}=\frac{f_{\theta_{\left(1\right)}}\left(a\right)}{f_{\theta_{\left(1\right)}}\left(b\right)}$$ (9)

where $f_{\theta_{\left(1\right)}}\left(a\right)$ is the marginal distribution evaluated in *a*. If this condition is met, then the continuous posterior distribution can be represented correctly using a discrete distribution (Bertsekas and Tsitsiklis, 2008). This is possible in any PMF because $w_{\left(1\right)}^{1}$ and $w_{\left(1\right)}^{2}$ are always normalized by the same denominator as in (3), so their ratio is constant.

In subset *θ*_(1)_, the weights are determined by Eq. 7, so they all directly follow

$$w_{\left(1\right)}^{i}∼f_{\theta_{\left(1\right)}}\;\forall i\in \left(1,N\right)$$ (10)

regardless of how many particles are used in the filter. It follows from (10) that a single particle with value *θ*_(1)_ = *a* is sufficient to sample directly the value of the marginal posterior distribution $f_{\theta_{\left(1\right)}}\left(a\right)$ . Therefore, (9) holds and we guarantee that the marginal is represented correctly.

Instead, in the PF case, the weights of particles represent a joint distribution $f_{\theta }=f_{\theta_{\left(1\right)},\theta_{\left(2\right)}}$ of the entire set of states. Due to their independence, the joint distribution can be represented as the product of every marginal distribution $f_{\theta_{\left(1\right)}}$ and $f_{\theta_{\left(2\right)}}$ (Bertsekas and Tsitsiklis, 2008), and we obtain that

$$w=w_{\left(1\right)}w_{\left(2\right)}∼f_{\theta_{\left(1\right)}}f_{\theta_{\left(2\right)}}=f_{\theta_{\left(1\right)},\theta_{\left(2\right)}}.$$ (11)

For this reason, if we want to derive an estimation of the marginal distribution of *θ*_(1)_ from the joint distribution, the influence from *θ*_(2)_ needs to be eliminated. In order to do that, we start by defining a set of particles *i*_*a*_ = 1, … , *M*_1_ that respect the condition of *θ*_(1)_ = *a*. We want to obtain the marginal weight, denoted by the hat, by sampling at that value ${\hat{w}}_{\left(1\right)}^{\theta_{\left(1\right)}=a}$ for the first subset *θ*_(1)_ from the total space set ***θ***. This means that we need to average the weight of all particles which belong to the set *i*_*a*_. This can be written as

$${\hat{w}}^{\theta_{\left(1\right)}=a}=\frac{1}{M_{1}}\overset{M_{1}}{\underset{i=1}{\sum }}w^{i_{a}}.$$ (12)

Because of the left side of (11), we can rewrite it as

$${\hat{w}}^{\theta_{\left(1\right)}=a}=\frac{1}{M_{1}}\overset{M_{1}}{\underset{i=1}{\sum }}w_{\left(1\right)}^{i_{a}}w_{\left(2\right)}^{i_{a}}.$$ (13)

Since all particles follow $\theta_{\left(1\right)}^{i}=a$ , then the first weight is the same for all and it can be taken out of the summation as

$${\hat{w}}^{\theta_{\left(1\right)}=a}=w_{\left(1\right)}^{\theta_{\left(1\right)}=a}\frac{1}{M_{1}}\overset{M_{1}}{\underset{i=1}{\sum }}w_{\left(2\right)}^{i_{a}}.$$ (14)

We know from (10) that $w_{\left(1\right)}^{\theta_{\left(1\right)}=a}$ is a sampling directly from $f_{\theta_{\left(1\right)}}\left(a\right)$ . Hence in (14), the approximation ${\hat{w}}^{\theta_{\left(1\right)}=a}$ equals to the true value $w_{\left(1\right)}^{\theta_{\left(1\right)}=a}$ times a scaling factor. The latter is influenced by the particular values of $w_{\left(2\right)}^{i_{a}}$ , which in turn depends on the values of *θ*_(2)_ of the particles in the set *i*_*a*_. Notice that this scaling value does not necessarily have to be equal to 1, as all weights are then normalized by a common factor. Instead, we want the average of $w_{\left(2\right)}^{i_{a}}$ , to be equal to the average of the entire set $w_{\left(2\right)}^{i}$ . Otherwise, approximations at different values of the marginal distribution of *θ*_(1)_ are multiplied by different scaling factors, leading to distortion. This effect is mitigated as *N* increases.

Using the same inference for the other particle ***θ*** ^***2***^, we define the set *i*_*b*_ = 1, … , *M*_2_ which satisfies *θ*_1)_ = *b*. Then, the marginal weight for *θ*_1)_ = *b* can be obtained as

$${\hat{w}}^{\theta_{\left(1\right)}=b}=w_{\left(1\right)}^{\theta_{\left(1\right)}=b}\frac{1}{M_{2}}\overset{M_{2}}{\underset{i=1}{\sum }}w_{\left(2\right)}^{i_{b}}.$$ (15)

Therefore,

$$\frac{{\hat{w}}^{\theta_{\left(1\right)}=a}}{{\hat{w}}^{\theta_{\left(1\right)}=b}}=\frac{w_{\left(1\right)}^{i=a}}{w_{\left(1\right)}^{i=b}}\frac{\frac{1}{M_{1}}\overset{M_{1}}{\underset{i=1}{\sum }}w_{\left(2\right)}^{i_{a}}}{\frac{1}{M_{2}}\overset{M_{2}}{\underset{i=1}{\sum }}w_{\left(2\right)}^{i_{b}}}.$$ (16)

Because the states *θ*_1_ and *θ*_2_ are independent, for any given *i*, the particle weights $w_{\left(2\right)}^{i}$ follow the same distribution with mean *μ* and variance *σ* ^2^. Applying the central limit theorem, the distribution containing $\frac{1}{M_{2}}\sum_{i}^{M_{2}}w_{\left(2\right)}^{i_{b}}$ approaches a normal distribution with mean *μ* and variance *σ* ^2^/*M*_2_ with the increasing of *M*_2_ (Bertsekas and Tsitsiklis, 2008). Therefore, only with a large number of total particles *N*, which also implies large *M*_1_ and *M*_2_, both $\frac{1}{M_{1}}\sum_{i}^{M_{1}}w_{\left(2\right)}^{i_{a}}$ and $\frac{1}{M_{2}}\sum_{i}^{M_{2}}w_{\left(2\right)}^{i_{b}}$ will converge to *μ*. Then, it follows that $\frac{{\hat{w}}_{\left(3\right)}^{\theta_{\left(1\right)}=a}}{{\hat{w}}_{\left(3\right)}^{\theta_{\left(1\right)}=b}}$ will converge to $\frac{f_{\theta_{1}}\left(a\right)}{f_{\theta_{1}}\left(b\right)}$ and the PDF can be represented without distortion.

In summary, given that *θ*_1_ and *θ*_2_ are independent, if we want to represent the marginal distribution of *θ*_1_ using a set of particles without distortion, the conventional PF needs more particles than the proposed MW-PF because it needs to eliminate the impact from *θ*_2_.

### 2.3 A numerical example

A numerical example is provided in order to show the appearance of a bias in the estimation given by the inaccurate approximation of the marginal posterior distribution when the number of particles is low and an inefficient weighting strategy is used.In order to show how an inaccurate approximation of the posterior distribution can produce errors on the estimate, a small numerical example is set up. We consider a single epoch simulation in which the state space vector is ***θ*** = [ *θ*_1_ *θ*_2_ ] with true values ***θ*** ^*T*^ = [ 4 4 ]. The input to the estimation problem is a set of two measurements **z** = [ z_1_ z_2_ ]. The observable-states function **h** that describes the relationship between measurements and states is in this case

$$z_{j}=\theta_{j}^{T}+v_{j}\;\forall j\in \left\{1,2\right\}$$ (17)

such that each measurement is a direct noisy observation of the corresponding state. The noise terms v_*j*_, in this example is assumed to be statistically distributed as zero mean Gaussian distributions with standard deviation *σ* = 2. This corresponds to the probability densities p∼N(0,2) used to compute the weights, as in (2). However, in order to focus on the error due to inaccurate sampling of the posterior probability, it is assumed that the realization on noise available at the targeted epoch are equal to zero, so that v_*j*_ = 0 *∀j* ∈ {1, 2}. We assume to have *N* = 3 particles with given values ***θ*** ^***1***^ = [ 4 6 ], ***θ*** ^***2***^ = [ 7 4 ], ***θ*** ^***3***^ = [ 1 2 ]. Moreover, all weights are initialized to $\frac{1}{N}=\frac{1}{3}$ .

First, the state estimate is computed through a conventional PF. We start by computing, for each input measurement, the difference the input value and the nominal one for each particle as in (1), which yields ${\bar{z}}^{1}=\left[\;0\;-2\;\right]$ , ${\bar{z}}^{2}=\left[\;-3\;0\;\right]$ and ${\bar{z}}^{3}=\left[\;3\;2\;\right]$ . These vectors are fed into 2) to obtain the weights, which are then normalized according to (3). A summary of the weights is reported in the third column of Table 1. The final estimate is obtained using (5) as

$$\begin{array}{cc} \hat{\theta } & =\overset{3}{\underset{i=1}{\sum }}w^{i}\theta^{i}=\left(0.538\cdot \left[\;4\;6\;\right]\right)+\left(0.288\cdot \left[\;7\;4\;\right]\right)+\left(0.175\cdot \left[\;1\;2\;\right]\right)\\ & =\left[\;4.340\;4.726\;\right]. \end{array}$$ (18)

**FIGURE 3:**
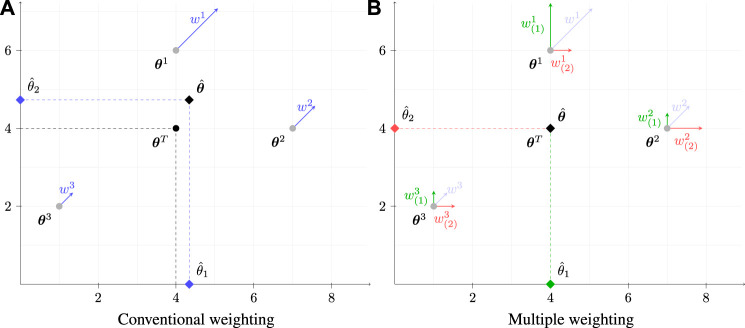
Comparison of a state-estimation using conventional **(A)** and the proposed MW **(B)** approaches applied to a simplistic two-dimensional scenario solved by means of a SIR PF with *N* =3 particles. Weights *w* ^*i*^, shown in **(A)**, are split in independent weights $w_{1,2}^{i}$ and normalized for each sub-state in **(B)**.

As it can be seen, the final estimation is biased w.r.t. the true value assumed for $\theta_{1}^{T}$ , $\theta_{2}^{T}$ , as also graphically depicted in Figure 3A.

**TABLE 1:** Summary of values and weights of particles.

	States		PF weights	MW-PF weights	
	*θ*_1_	*θ*_2_	*W*	*w*_(1)_	*w*_(2)_
***θ*** ^***1***^	4	6	0.538	0.606	0.274
***θ*** ^***2***^	7	4	0.288	0.197	0.452
***θ*** ^***3***^	1	2	0.175	0.197	0.274

When performing the estimation according to the proposed MW-PF, the subsets ***θ***_***1)***_ = [ *θ*_1_ ] and ***θ***_***2)***_ = [ *θ*_2_ ] have to be considered. In this case, only the first measurement contributes to ${\bar{z}}_{1}^{1}$ , which is used to obtain the first weight $w_{\left(1\right)}^{i}$ through 7) (with *M*_(1)_ = 1). The same procedure can be followed to obtain the weights $w_{\left(2\right)}^{i}$ from ${\bar{z}}_{2}^{1}$ . After normalisation, the weights take the values reported in the last two columns of Table 1. The final estimate is obtained using (8) as

$$\begin{array}{c} {\hat{\theta }}_{\left(1\right)}=\overset{3}{\underset{i=1}{\sum }}w_{\left(1\right)}^{i}\theta_{\left(1\right)}^{i}=\left(0.606\cdot 4\right)+\left(0.197\cdot 7\right)+\left(0.197\cdot 1\right)=4\\ {\hat{\theta }}_{\left(2\right)}=\overset{3}{\underset{i=1}{\sum }}w_{\left(2\right)}^{i}\theta_{\left(2\right)}^{i}=\left(0.274\cdot 6\right)+\left(0.452\cdot 4\right)+\left(0.274\cdot 2\right)=4 \end{array}$$ (19)

and so there is no error on the estimate, as depicted in Figure 3B.

Because of the independence, the marginal posterior density of the first state is described by a Gaussian distribution $f_{\theta_{\left(1\right)}}∼N\left(\mu ,\sigma \right)$ which is symmetric around the mean *μ* = 4. Therefore,

$$\frac{f_{\theta_{\left(1\right)}}\left(\mu +\epsilon \right)}{f_{\theta_{\left(1\right)}}\left(\mu -\epsilon \right)}=1$$ (20)

is always true for any given value *ϵ*. Duo to our assumptions, we notice that particle states $\theta_{1}^{2}=7$ and $\theta_{1}^{3}=1$ are indeed symmetric around *μ* = 4. So we compute the ratio of their weights for MW-PF and PF cases as

$$\begin{array}{cc} & \frac{w_{\left(1\right)}^{2}}{w_{\left(1\right)}^{3}}=\frac{0.197}{0.197}=1\\ & \frac{w^{2}}{w^{3}}=\frac{0.288}{0.175}=1.648 \end{array}$$ (21)

and notice that it is not equal to one for the latter. Since in this example *N* is not large, the influence of the second state *θ*_2_ is not averaged out, and the PF is not able to accurately represent the marginal posterior density, leading to an error on the estimation.

### 2.4 Application to GNSS positioning

This section is dedicated to the implementation of the proposed MW-PF to precise state estimation in GNSS receivers. In Section 1, it was mentioned how the main advantage of PF is the ability to handle non-linear models and non-Gaussian probability densities without loss of performance. While these conditions are mainly encountered in scenarios when GNSS is integrated with external measurement, our proposed method can be applied regardless of the scenario and there is no need to focus on a specific one. Therefore, for the sake of the brevity of our discussion and simplicity of the notation, we present here an implementation based on stand-alone GNSS.

In this scenario, there are two types of measurements that GNSS receivers can obtain by receiving and processing the navigation signals broadcasted by satellites. Namely, *pseudoranges* and *range rates* (which are related to Doppler shift). In this study, we employ zero-mean Gaussian distribution as probability densities of measurement errors. Even tough this choice could be sub-optimal in some scenarios, we expect both filter architectures would be equally penalized by this choice so that any comparison remains fair.

#### 2.4.1 GNSS measurements model and state estimation

A generic GNSS receiver is tasked with the estimation of the following state space vector

$$\theta =\left[\;\underset{\theta_{\left(1\right)}}{\underset{︸}{x\;y\;z\;b}}\;\underset{\theta_{\left(2\right)}}{\underset{︸}{\dot{x}\;\dot{y}\;\dot{z}\;\dot{b}}}\;\right]$$ (22)

where the variables [ *x y z* ] refers to the spatial coordinates in a given Cartesian reference system, and $\left[\;\dot{x}\;\;\dot{y}\;\;\dot{z}\;\right]$ to the axial velocity components, while *b* and $\dot{b}$ are respectively the bias and drift of the local clock. In the MW approach, the two subsets of the state space vector are denoted with ***θ***_(1)_ and ***θ***_(2)_.

The first class of observables, namely pseudoranges, is defined as

$$\rho_{s}=\sqrt{{\left(x_{s}-x\right)}^{2}+{\left(y_{s}-y\right)}^{2}+{\left(z_{s}-z\right)}^{2}}+b.$$ (23)

where subscript *s* denotes a generic satellite. The pseudorange equation consists of the Euclidean distance between satellite *s* and the receiver, plus the clock bias.

In order to introduce the second class of measurements, range rates, we first define the differential vector quantities of position and velocity

$$\begin{array}{c} \Delta p=\left[\;\left(x_{s}-x\right)\;\left(y_{s}-y\right)\;\left(z_{s}-z\right)\;\right]\\ \Delta v=\left[\;\left({\dot{x}}_{s}-\dot{x}\right)\;\left({\dot{y}}_{s}-\dot{y}\right)\;\left({\dot{z}}_{s}-\dot{z}\right)\;\right] \end{array}$$ (24)

so that range rates can then be expressed as

$$\Delta \rho_{s}=\left(\Delta v\cdot \frac{\Delta p^{T}}{\left\|\Delta p\right\|}\right)+\dot{b}.$$ (25)

In the investigated application, pseudorange measurements do not provide any knowledge about the receiver velocity, but only about its position and clock bias, as can be seen from (23). As a consequence, using range information to compute a weight that also contributes to the estimation of the velocity leads to a sub-optimal use of particles.

On the other hand, it is worth noting that from (25) the range rate measurement has a limited dependency on the particle position. A key assumption introduced here is that the difference in position between the particles has a negligible contribution to the computation of the nominal range rate. In other words, we assume that if particles all had the same velocity, they would measure the same range rate. Since the distance between satellites and particle is much greater than the distance between any two particles, all the vectors Δ ***p*** pointing from the particles to the satellite can be considered parallel to each other. Eq. 25 computes the normalized projection of the relative velocity Δ ***v*** on vector Δ ***p***. Since the latter contribution is approximated to be the same for all particles, then the range rate measurement depends only on the velocity and clock drift of the particle.

This key assumption allows to perform a split of the input measurements as

$$z=\left[\;\underset{z_{\left(1\right)}}{\underset{︸}{\rho_{1}\;\ldots \;\rho_{M_{\left(1\right)}}}}\;\underset{z_{\left(2\right)}}{\underset{︸}{\Delta \rho_{1}\;\ldots \;\Delta \rho_{M_{\left(2\right)}}}}\;\right]$$ (26)

where subscripts *M*_(1)_ and *M*_(2)_ are the number of available pseudoranges and range rates measurements respectively. Since in general, for each visible satellite, it is possible to obtain one measurement for each of the two classes, we consider that *M*_(1)_ = *M*_(2)_.

**FIGURE 4:**
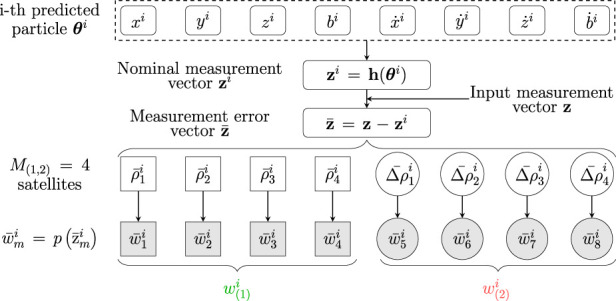
Weight computation stage of the proposed MW-PF architecture, based on two subsets of observables according to (7).

Figure 4 provides a block scheme of the computation of the two weights in the MW-PF architecture, similarly to how it is described in Section 1.1.2. In particular, we denote with ${\bar{w}}_{m}^{i}$ the output of the probability densities which are being multiplied in (7). It is clear from the scheme how pseudoranges only contribute to the computation of the first weight, and vice versa range rates only to compute the second weight.

## 3 Results

The experiment data was collected using the Navigation Constellation Simulator (NCS) simulator, a GNSS signal simulation and generation system. The ephemeris and observation data, including pseudoranges and Doppler shifts was stored in RINEX format. All the observations are of the Global Positioning System (GPS) constellation with the L1 C/A signal. To simulate noise, we add noise via ionosphere noise model with the standard deviation of 2 and 1 m for pseudoranges in the static and dynamic scenarios, respectively, and 1 Hz for Doppler shifts in both scenarios. Input measurements are collected at a rate of 10 Hz. To validate our proposed algorithm, both static and dynamic scenarios were built.

### 3.1 Static scenario

**FIGURE 5:**
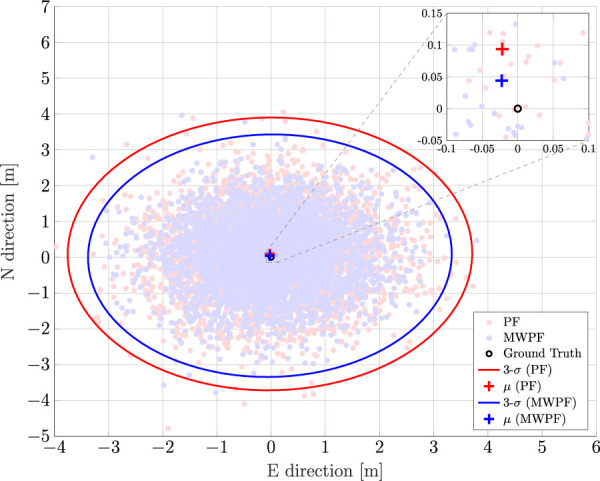
Comparison of PF and MW-PF solutions (in east-north reference frame) applied to position estimation of a static GNSS receiver for *N* =2000. Mean value of the estimate and 3-*σ* uncertainty in the form of error ellipses are also depicted for the two distributions. The ground truth is located in (0,0).

Although Bayesian estimation is primarily exploited for kinematic state estimation, accurate static state estimation is still of interest as it can temporarily occur in any real trajectory. Moreover, it can be an interesting baseline assessment for the performance of any positioning algorithm. Therefore, an experiment involving a static position estimation is performed first. Figure 5 plots all the positioning solutions obtained with the PF and MW-PF for all epochs of the simulations. The plot represents the East-North plane of a local East-North-Up (ENU) reference system, with the ground truth in its center. To better visually display the difference in performance between the two implementations of the PF, we chose for this plot the solutions when a low number of particles is used (*N* = 2000), and the improvement given by our proposed method is more stark.

**FIGURE 6:**
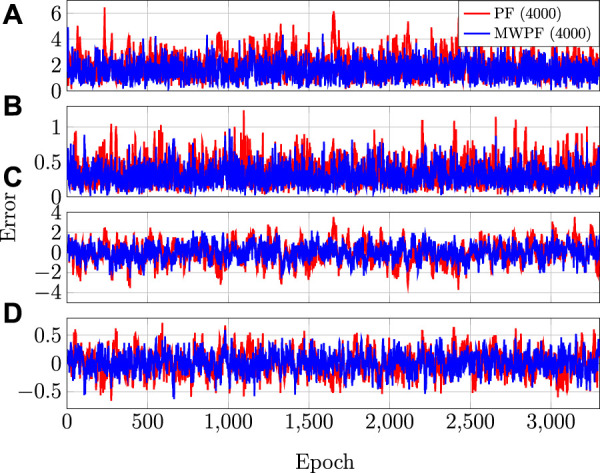
**(A)** RSE on position **(B)** RSE on velocity **(C)** Clock bias error **(D)** Clock drift error.

The errors on all the state variables over time is instead displayed in Figure 6 for *N* = 4000. As it can be seen, the MW-PF is more accurate in the estimation of all the state variables.

**FIGURE 7:**
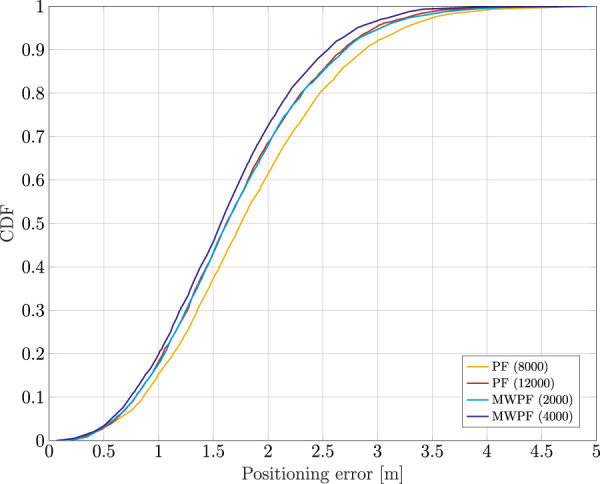
Comparison of the CDF of the positioning error of a static receiver using PF and MW-PF solutions with different numbers of particles.

Eventually, Figure 7 shows the CDF of the positioning error for both algorithms, tested for some selected number of particles. In reality, more values were tested but were in the end omitted for the sake of clarity of the plot. In particular for the MW-PF, going beyond *N* = 4000, the performance did not improve any further. For the PF instead, as it can be inferred from the plot, for values lower than *N* = 8000 the performance degraded very quickly. Instead, values above *N* = 12000 were not tested as the simulations became increasingly time consuming. More details on the computational complexities will be given later, but for now it is interesting to notice how the performance of PF for *N* = 12000 is very close to that of MW-PF for *N* = 2000. The important take-away from this observation is that MW-PF can reach the same target accuracy with a significant reduction of the computational load. On the other hand, for a fixed available (and reasonable, meaning *N* is not too large) computational effort, the MW-PF can outperform the PF in terms of accuracy of the positioning solution.

### 3.2 Dynamic scenario

**FIGURE 8:**
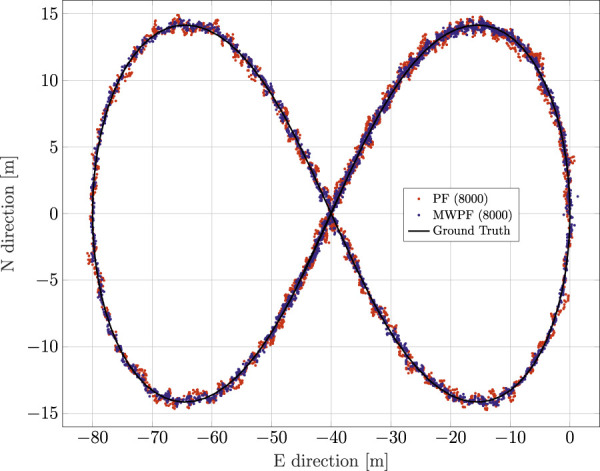
Comparison of PF and MW-PF solutions (in east-north reference frame) applied to position estimation of a dynamic GNSS receiver.

For a second assessment, an artificial dynamic trace is used with the shape of a Bernoulli lemniscate, as can be seen in Figure 8, which also displays the positioning solutions for both algorithms (*N* = 8000). The moving target performs roughly one loop of the track during the simulations. By comparing the positioning solutions of Figure 8 it can be seen how, especially in some parts of the trajectory, the MW-PF solution is consistently closer to the ground truth.

**FIGURE 9:**
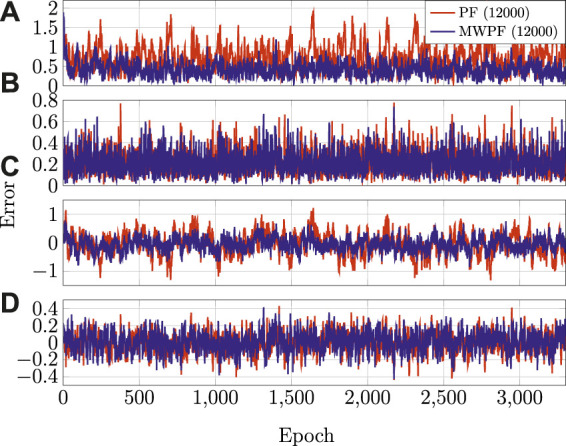
**(A)** RSE on Horizontal position **(B)** RSE on Horizontal velocity **(C)** Clock bias error **(D)** Clock drift error.

**FIGURE 10:**
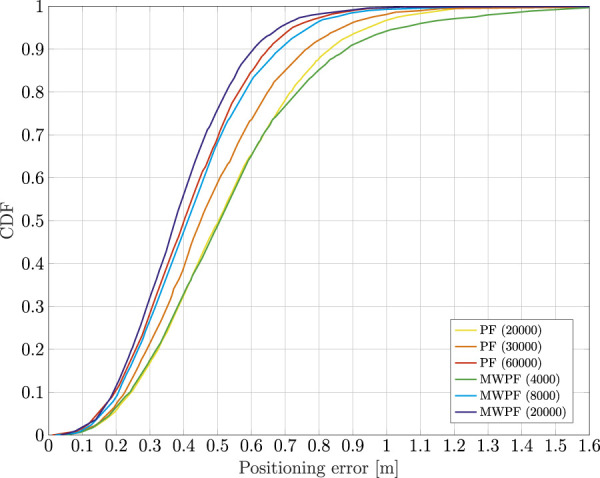
Comparison of the CDF of the positioning error of a dynamic receiver using PF and MW-PF solutions with different numbers of particles.

As done for the static case, the error on the state variables of interest is shown in Figure 9. Once again, a deliberate choice of plotting the errors of the two algorithms for a lower number of particles was made in order to emphasize the difference in their performance. In particular, it is interesting to notice from subplots 1) and 3) how in this scenario the improvement in accuracy given by MW-PF is larger for the estimation of position and clock bias. This difference was not as stark when comparing the same errors of the static scenario. This phenomenon can be quantified by looking at Tables 2, 3 which provide a summary of the two tests. The improvement column refers to the percentage decrease in Root Mean Squared Error (RMSE) when employing MW-PF instead of PF. We remind that from (22), that position and clock bias are the variables chosen to form the first sub-vector, since pseudorange measurements provide information about those state, as can be seen from (23). This results suggests that, when the target is in a dynamic state, splitting the estimation of position and clock bias with their respective derivatives, the gain in estimation accuracy is larger for the former. The CDF of the positioning solution of both algorithms is shown in Figure 10. We selected the results for some specific number of particles in order to not overcrowd the plot. The take-away from this results is similar to what observed for the static scenario, which is that MW-PF can reach the same accuracy of PF with a reduced number of particles. Finally, 11 shows the error at the 90th percentile of the CDF for both algorithms and different values of particles. We wanted to investigate whether by further increasing *N* for PF, its performance would eventually reach or even surpass that of MW-PF. The last value we tested was *N* = 60000 since simulations eventually became too long to continue. This last test yielded a 90th percentile error of 0.650 against one of 0.607 for MW-PF at *N* = 20000. The conclusion is that even when *N* is extremely large, the performance of PF doe not fully converge to that of MW-PF, suggesting that some small residual additional errors remain due to the sub-optimal sampling of the algorithm.

**TABLE 2:** Comparison of the static scenario between MW-PF and PF (both at *N* =4000) in terms of RMSE on the state variables.

State	PF	MW-PF	Improvement (%)
3D Position	2.084	1.613	22.6
3D Velocity	0.371	0.304	18.1
Clock Bias	1.089	0.766	29.7
Clock Drift	0.217	0.173	20.3

**TABLE 3:** Comparison of the dynamic scenario between MW-PF and PF (both at *N* =12000) in terms of RMSE on the state variables.

State	PF	MW-PF	Improvement (%)
3D Position	0.724	0.418	42.3
3D Velocity	0.224	0.219	2.2
Clock Bias	0.391	0.240	38.6
Clock Drift	0.120	0.116	3.3

**FIGURE 11:**
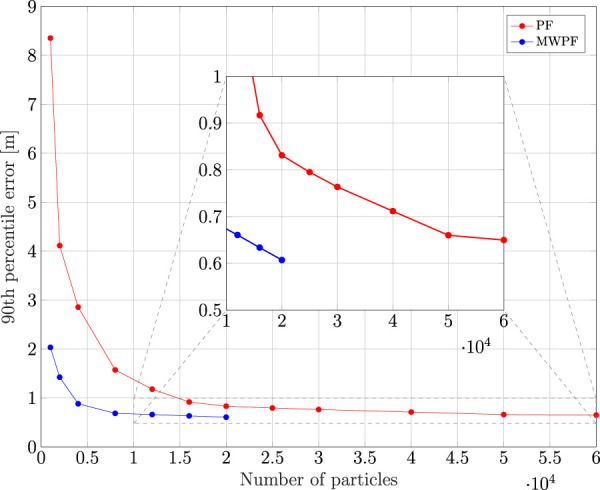
Comparison of 90th percentile error of PF and MW-PF solutions with different numbers of particles.

Given the results from Figure 11 for MW-PF, we identify values of *N* between 4000 and 12000 as possible good working points in terms of trade-off between computational load and accuracy.

### 3.3 Computational complexity

Since the two algorithms presented in these results present some differences in their code and implementation, a summery of their execution times is given in order to give a fair comparison between the two. The results are reported in Table 4 for some values of *N*. By fixing any *N*, the run time of MW-PF is slightly longer than PF as expected, since some computations and checks are performed twice. Overall, this increase is not large and is mostly offset by the fact that the MW-PF implementation can reach the same accuracy with fewer particles.

**TABLE 4:** Comparison of the simulation run times in seconds between PF and MW-PF to solve 3000 epochs of PVT.

***N***	PF	MW-PF
1000	6.26	6.40
2000	7.98	8.05
4000	11.15	11.30
8000	15.00	15.58

It is important to stress that the times reported here are given simply in order to provide a comparison between the two algorithms, rather than to give a thorough investigation of the computational complexities of PF. In fact, no parallel optimization has been implemented (although we anticipate to do so in the future), despite some heavy computations of PFs could be implemented this way, leading to a reduction of the run times.

## 4 Discussion

This paper has presented a technique, named MW, to exploit the information diversity of input measurements in order to achieve a more accurate sampling of the posterior distribution with fewer particles. Despite being applied to GNSS here, MW can be generalized to be exploited in other types of state estimation problems with minimum modifications of the PF routine. While in the investigated application the state vector was split in two subsets, any number of such subsets is possible in principle, according to the relationship between measurements and states in the system of interest. Along with its description, the paper also presented a mathematical derivation to support the technique, as well as a simplified and intuitive example to show the advantage of the proposed method.

An extensive simulation campaign has been performed, including both static and dynamic scenarios. Results show that, for both cases, MW-PF provides better performance in terms of accuracy, especially when a low number of particles is used. In particular, the same accuracy obtained through PF can be reached with MW-PF with as low as one fifth of the particles. On the other hand, for the same *N* = 12000 in the dynamic scenario, MW-PF can provide an improvement of over 40% in terms of positioning error.

Indeed, when sampling over multiple weights, each particle retains information about the likelihood of each subset of states, rather than an overall likelihood across all states. Since each particles holds more information about the posterior, an accurate representation can be obtained with fewer particles. In fact, the proposed MW-PF is able to mitigate the main drawback of SMC methods w.r.t. to KF. Furthermore, it should be added that since the resampling stage is performed independently on the subsets, another advantage of the MW approach is that this step can be implemented in parallel in a straight-forward manner, thus possibly further reducing its run time.

MW-PF is a propedeutic concept to bridge traditional Bayesian estimation and AI approaches. The proposed architecture naturally requires an automated subspace identification through a state-measurements relationship for intelligent management of the computational resources. Future works may address AI solutions to automate the positioning problem analysis or the estimation problem to a large extent.

## Data Availability

The raw data supporting the conclusion of this article will be made available by the authors, without undue reservation.
